# Defining standards for modelling the biomechanics of the foot and ankle: a systematic review

**DOI:** 10.1186/1757-1146-4-S1-O9

**Published:** 2011-05-20

**Authors:** Chris Bishop, Gunther Paul, Dominic Thewlis

**Affiliations:** 1 School of Health Science, University of South Australia, Adelaide, Australia; 2 Mawson Institute, University of South Australia, Mawson Lakes, Australia

## Background

The complex interaction of the bones of the foot has been explored in detail in recent years, which has led to the acknowledgement in the biomechanics community that the foot can no longer be considered as a single rigid segment. With the advance of motion analysis technology it has become possible to quantify the biomechanics of simplified units or segments that make up the foot. Advances in technology coupled with reducing hardware prices has resulted in the uptake of more advanced tools available for clinical gait analysis. The increased use of these techniques in clinical practice requires defined standards for modelling and reporting of foot and ankle kinematics. This systematic review aims to provide a critical appraisal of commonly used foot and ankle marker sets designed to assess kinematics and thus provide a theoretical background for the development of modelling standards.

## Methods

An electronic database search was performed in March 2010. The search strategy used was “foot model* AND human* AND kinematic* AND (gait* OR ergonomic* OR automotive*)”. The secondary snowball method was applied to identify literature not identified during the electronic database searching process. Inclusion/exclusion criteria were applied by one reviewer (CB). Data was extracted based on standardised protocol. The quality of studies was assessed by two reviewers (CB and DT) based on a custom built assessment tool.

## Results

The initial electronic database search identified 287 articles. 224 were excluded on review of title and abstract only. Inclusion/exclusion criteria excluded 48 articles. A secondary snowball search identified a further 4 articles. 19 original articles were included in the final review.

## Conclusions

The results of this systematic review suggests that the foot should either be modelled as a single rigid segment to analyse ankle joint kinematics or at least three segments to properly define hind-, mid- and forefoot kinematics. Future analysis of interventions such as in-shoe wedging, foot orthoses and footwear design requires such a model. However, health professionals must appreciate that the complexity of the marker set used should be indicative of the complexity of the analytical question being asked.

